# Nucleosynthetic constraints on the origin of Bennu and CI-like asteroids

**DOI:** 10.1126/sciadv.aei9107

**Published:** 2026-09-23

**Authors:** Maria Schönbächler, Mattias Ek, Manuela A. Fehr, Katarzyna M. Liszewska, Lara A. E. Meyer, Miriam Rüfenacht, James M. J. Ball, Paul Frossard, Jan Render, Quinn R. Shollenberger, Greg A. Brennecka, Thomas S. Kruijer, Josh Wimpenny, Martin Bizzarro, Jessica J. Barnes, Ann N. Nguyen

**Affiliations:** ^1^ Institute of Geochemistry and Petrology, ETH Zurich, Zurich, Switzerland.; ^2^ Lawrence Livermore National Laboratory, Livermore, CA, USA.; ^3^ Center for Star and Planet Formation; Globe Institute, University of Copenhagen, Copenhagen, Denmark.; ^4^ Lunar and Planetary Laboratory, University of Arizona, Tucson, AZ, USA.; ^5^ ARES, NASA Johnson Space Center, Houston, TX, USA.

## Abstract

Analyses of returned samples from asteroids Bennu and Ryugu reveal notable similarities in chemical and isotopic compositions to Ivuna-type (CI) carbonaceous chondrites, prompting reassessment of their origin. We present nucleosynthetic iron (Fe), titanium (Ti), and chromium (Cr) isotope data for Bennu. Minor Cr isotope variations among aliquots likely reflect parent body aqueous alteration, whereas Fe and Ti isotope data are homogeneous and indistinguishable from those of Ryugu and CI chondrites at bulk (>20 milligrams) scale. These results demonstrate that CI-like bodies accreted from a common dust reservoir with affinities to both inner (noncarbonaceous) and outer (carbonaceous) Solar System materials. Combined isotopic, petrographic, and chemical evidence suggests formation near the water ice line inside proto-Jupiter’s orbit, where Jupiter’s growth restricted the inward drift of larger particles, whereas efficient mixing of fine-grained dust produced bodies with near-solar compositions.

## INTRODUCTION

C-type asteroids comprise ∼75% of known main-belt asteroids and are estimated to account for ∼40 to 50% of the belt’s total mass (*1*). Two recent sample return missions—NASA’s OSIRIS-REx and JAXA’s Hayabusa2—returned samples of two subgroups of C-type asteroids: the B-type asteroid Bennu and Cb-type Ryugu (*2*, *3*). Both bodies are rubble pile asteroids, consisting of fragments of one or more larger parent bodies that were collisionally disrupted (*4*). Despite their distinct spectral characteristics, analyses of the returned material from both asteroids revealed notable chemical and isotopic similarities to Ivuna-type (CI) carbonaceous chondrite (CC) meteorites (*5*, *6*). In addition, evidence from meteorite falls, such as asteroid 2008 TC3 (Almahata Sitta meteorite), hints at more abundant fine-grained, hydrated CI-related material in asteroids (*1*, *7*) than previously estimated based on the scarcity of CI chondrites in meteorite collections [10 meteorites, 0.0127% of total record (*8*)]. This likely reflects the friability of the material, which is prone to destruction in space and, in particular, during atmospheric entry on Earth (*9*). Samples from Bennu, Ryugu, and CI chondrites are the only known materials available for laboratory study that closely reflect solar elemental abundances as determined from the solar photosphere (*10*), with few exceptions (e.g., highly volatile elements such as N, O, and noble gases). Because the Sun contains more than 99% of the Solar System’s mass, its composition serves as a unique proxy for the average Solar System composition and provides a critical baseline for planet formation models including the delivery of volatiles to Earth.

The near solar elemental abundance of CI meteorites is particularly remarkable given their petrological complexity. Bennu samples are dominated by fine-grained, intimately mixed assemblages of diverse components (nanometer to micrometer on average). Ices originally incorporated into the parent asteroid were melted and subsequently altered the surrounding material to varying degrees through aqueous alteration, leading to the formation of phyllosilicates (80 vol %) with minor amounts of magnetite, sulfides, carbonates, and phosphates (*11*). Nevertheless, some Bennu samples still host anhydrous silicates, which are refractory solids that crystallized or formed near the early Sun, along with organic matter from the outer Solar System or the parent molecular cloud, in addition to presolar grains bearing extreme isotopic compositions produced by multiple earlier generations of stars (*5*).

Linking asteroids to known meteorite groups remains challenging (*1*). However, high-precision nucleosynthetic isotope analyses of returned samples offer a unique opportunity to establish genetic relationships between meteorites and their parent bodies (*5*, *6*). Planetary bodies are characterized by distinct nucleosynthetic signatures that can act as identifiers (*12*), although overlap may occur among genetically related bodies [e.g., the Earth-Moon system (*13*)]. These nucleosynthetic compositions record the heterogeneous distribution of isotopically distinct material in the protoplanetary disk, carrying specific isotopic signatures originating from different stellar formation environments. They therefore provide key insights into the formation region of asteroidal parent bodies.

Initial bulk Ti and O isotope compositions of Bennu aggregate samples revealed a genetic relationship with CIs and asteroid Ryugu while being distinct from other meteorite classes (*5*). Here, we report nucleosynthetic Ti, Cr, and Fe isotope data for multiple bulk Bennu samples of varying sizes and morphological particle types, as well as for selected CCs, to obtain constraints on the formation region of Bennu’s parent asteroid. Our data also provide constraints on the level of localized parent body heterogeneity because nucleosynthetic compositions can undergo modification during aqueous alteration on the parent body (*14*), leading to small-scale variations in chondrites [e.g., for Cr and Os (*6*)], depending on the robustness of their isotopically anomalous carrier phases.

## RESULTS

### Chromium isotope parent body heterogeneity in Bennu, Ryugu, and CI chondrites

**Fig. 1.**
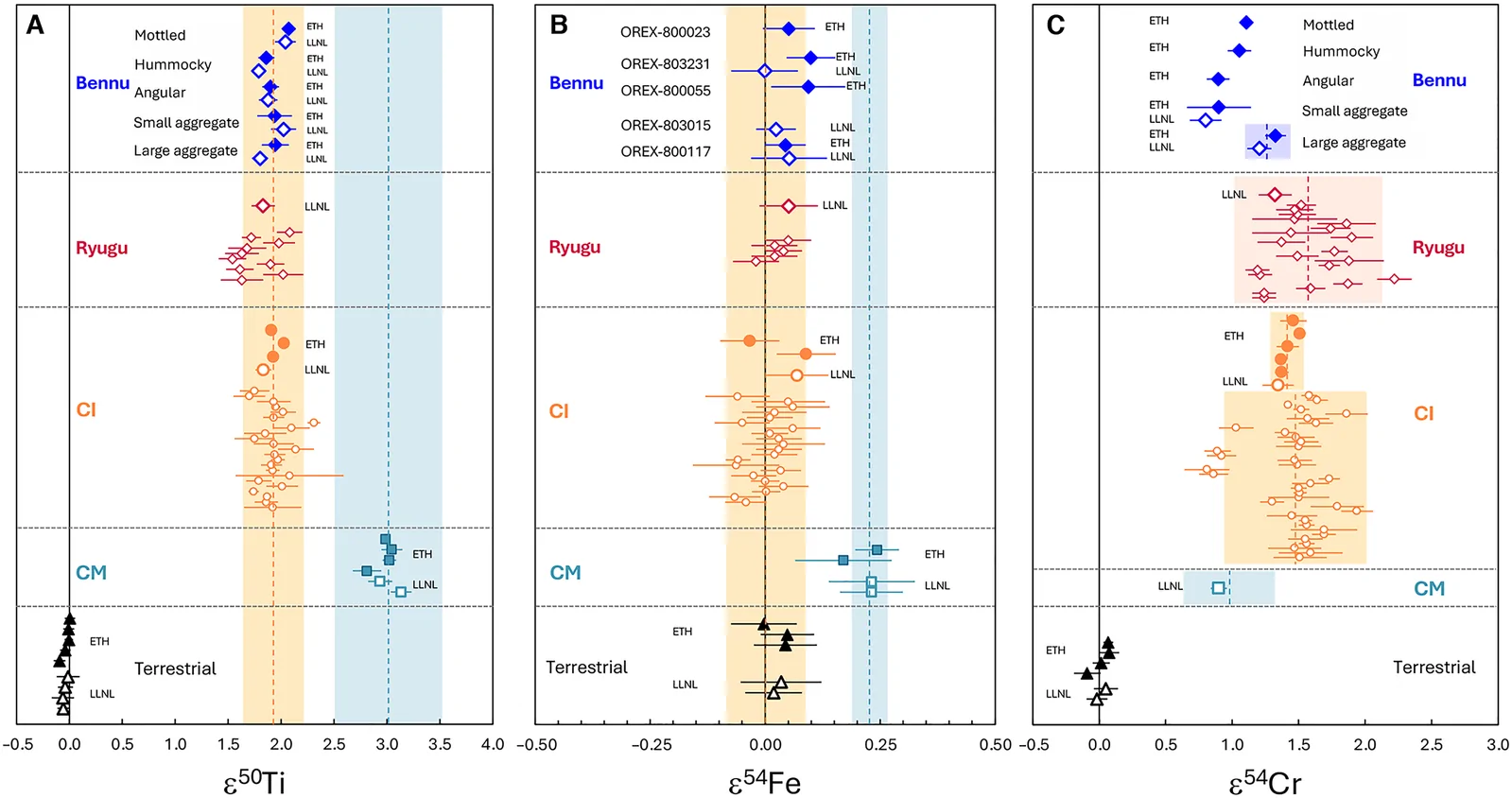
Nucleosynthetic isotope systematics for Solar System materials. (**A**) Ti, (**B**) Fe, and (**C**) Cr isotope data for Bennu, Ryugu, CI, and CM chondrites and terrestrial samples. Orange lines and bars represent the average and 2 SD of CI chondrite data, whereas turquoise refer to CM data. Dark blue bar and line denote OREX-800117 (large aggregate); red Ryugu data (C). Data labeled with ETH (closed large symbols) and LLNL (open large symbols) are from Table 1. For literature data (small open symbols), see data S1 to S4.

Titanium, Cr, and Fe isotope data are reported for five Bennu aliquots (Fig. 1 and Table 1), including the angular, hummocky, and mottled particle types [as defined by the Bennu particle-type petrography framework (*11*)] as well as two homogenized powders of aggregate material, the latter two with distinct powdered masses of 20.7 and 1288 mg (Table 1). The data are given in epsilon (ε) notation, which is the deviation of a given isotopic ratio from a terrestrial standard in parts per 10^4^. Sample aliquots were analyzed in two independent laboratories (ETH and LLNL; Materials and Methods). Results from aliquots analyzed in both laboratories agree within analytical uncertainty (Fig. 1 and Table 1), confirming the robustness of the dataset.

**Table 1.** Bulk Ti, Cr, and Fe isotope compositions of Bennu, meteorite, and terrestrial samples.

Sample	Type, class	mg*	mg†	Laboratory	*n* ‡	ε^46^Ti§	ε^48^Ti§	ε^50^Ti§	*n* ‡	ε^54^Fe§	ε^58^Fe§	*n* ‡	ε^53^Cr§	ε^54^Cr§
OREX-800055-101¶	Angular	69	69	ETH	10	0.23 ± 0.07	−0.05 ± 0.02	1.90 ± 0.08	15	0.09 ± 0.08	0.13 ± 0.11	11	0.34 ± 0.06	0.89 ± 0.09
OREX-800055-2¶	Angular	69	69	LLNL	12	0.34 ± 0.11	−0.02 ± 0.04	1.88 ± 0.09						
OREX-803231-102#	Hummocky	37	30.6	ETH	7	0.29 ± 0.07	0.00 ± 0.05	1.86 ± 0.08	25	0.10 ± 0.05	0.05 ± 0.09	5	0.31 ± 0.03	1.05 ± 0.09
OREX-803231-100#	Hummocky	37	30.6	LLNL	5	0.28 ± 0.05	0.02 ± 0.09	1.79 ± 0.05	14	0.00 ± 0.07	0.06 ± 0.17			
OREX-800023-111**	Mottled	135	129	ETH	19	0.40 ± 0.05	−0.10 ± 0.01	2.07 ± 0.04	10	0.05 ± 0.06	0.09 ± 0.19	8	0.25 ± 0.04	1.11 ± 0.05
OREX-800023-112**	Mottled	135	129	LLNL	6	0.27 ± 0.10	−0.05 ± 0.06	2.04 ± 0.10						
OREX-803015-100††,##	Aggregate	20.7	20.7	ETH	4	0.26 ± 0.18	0.03 ± 0.07	1.94 ± 0.16				2	0.20 ± 0.04	0.90 ± 0.24
OREX 803015-101††,##	Aggregate	20.7	20.7	LLNL	10	0.27 ± 0.08	−0.07 ± 0.06	2.02 ± 0.12	20	0.02 ± 0.04	0.08 ± 0.11	9	0.25 ± 0.05	0.80 ± 0.12
OREX-800117-110	Aggregate	1288	483	ETH	8	0.33 ± 0.06	0.01 ± 0.04	1.95 ± 0.13	25	0.04 ± 0.04	0.06 ± 0.14	10	0.20 ± 0.06	1.33 ± 0.08
OREX 800117-111	Aggregate	1288	464	LLNL	14	0.42 ± 0.12	−0.03 ± 0.04	1.80 ± 0.08	13	0.05 ± 0.08	0.05 ± 0.12	8	0.19 ± 0.05	1.20 ± 0.09
Orgueil	CI1			ETH	6	0.37 ± 0.07	0.03 ± 0.03	1.93 ± 0.05						
Orgueil	CI1			ETH	9	0.32 ± 0.09	0.00 ± 0.02	2.03 ± 0.05	20	0.09 ± 0.06	0.00 ± 0.14	6	0.13 ± 0.06	1.37 ± 0.05
Orgueil	CI1			ETH	16	0.36 ± 0.05	−0.02 ± 0.03	1.91 ± 0.04	10	−0.03 ± 0.06	0.06 ± 0.18	4	0.14 ± 0.04	1.37 ± 0.04
Orgueil***	CI1			ETH	9	0.31 ± 0.10	−0.02 ± 0.06	1.92 ± 0.06				5	0.18 ± 0.05	1.46 ± 0.10
Orgueil	CI1			LLNL	10	0.33 ± 0.11	−0.07 ± 0.05	1.83 ± 0.07	14	0.07 ± 0.07	0.02 ± 0.13	11	0.20 ± 0.04	1.35 ± 0.12
Ivuna PB***	CI1			ETH	9	0.31 ± 0.05	0.02 ± 0.03	1.97 ± 0.06				10	0.18 ± 0.05	1.42 ± 0.08
Ivuna HP***	CI1			ETH	7	0.39 ± 0.05	−0.02 ± 0.04	1.91 ± 0.10				6	0.19 ± 0.02	1.51 ± 0.04
Aguas Zarcas‡‡,##	CM2			ETH	5	0.69 ± 0.06	−0.05 ± 0.07	2.81 ± 0.13	13	0.17 ± 0.10	0.04 ± 0.18			
Aguas Zarcas‡‡	CM2			ETH	11	0.53 ± 0.08	−0.03 ± 0.03	3.02 ± 0.06						
Aguas Zarcas‡‡	CM2			ETH	12	0.58 ± 0.06	0.00 ± 0.03	3.05 ± 0.10	20	0.24 ± 0.05	0.05 ± 0.13			
Aguas Zarcas‡‡	CM2			LLNL	5	0.59 ± 0.08	−0.04 ± 0.06	2.94 ± 0.11	13	0.23 ± 0.09	−0.12 ± 0.20			
Jbilet Winselwan†††	CM2			ETH	21	0.50 ± 0.05	−0.03 ± 0.02	2.99 ± 0.04						
Murchison	CM			LLNL	11	0.59 ± 0.11	−0.04 ± 0.07	3.13 ± 0.10	13	0.23 ± 0.07	−0.11 ± 0.16	6	0.23 ± 0.07	0.90 ± 0.06
Calama 005	CO3			LLNL	6	0.45 ± 0.11	0.01 ± 0.05	2.69 ± 0.12	13	0.24 ± 0.08	−0.09 ± 0.14			
Allende	CV3			LLNL	11	0.82 ± 0.14	0.02 ± 0.06	4.31 ± 0.14	12	0.27 ± 0.07	0.09 ± 0.14	6	0.17 ± 0.06	0.93 ± 0.09
Tarda§§	C2-ung.			ETH	6	0.50 ± 0.08	−0.02 ± 0.04	2.40 ± 0.06	15	0.31 ± 0.09	0.07 ± 0.10	6	0.26 ± 0.08	1.22 ± 0.15
Tarda§§	C2-ung.			ETH	12	0.40 ± 0.05	−0.04 ± 0.02	2.40 ± 0.03	10	0.38 ± 0.08	−0.02 ± 0.12	5	0.27 ± 0.07	1.31 ± 0.12
Tarda	C2-ung.			LLNL	5	0.45 ± 0.11	−0.04 ± 0.07	2.65 ± 0.11	12	0.28 ± 0.06	−0.18 ± 0.17			
Pasamonte	Eucrite			ETH	9	−0.31 ± 0.04	−0.02 ± 0.02	−1.29 ± 0.07	14	0.21 ± 0.09	0.06 ± 0.14			
Estacado	H6			ETH								4	0.21 ± 0.06	−0.38 ± 0.06
BHVO-2##	Terrestrial			ETH	9	0.01 ± 0.06	0.01 ± 0.03	−0.09 ± 0.05						
BHVO-2¶¶	Terrestrial			ETH	11	0.00 ± 0.04	0.05 ± 0.03	−0.04 ± 0.05	15	0.04 ± 0.07	−0.09 ± 0.09			
BHVO-2¶¶	Terrestrial			ETH	10	−0.08 ± 0.04	−0.01 ± 0.03	−0.01 ± 0.04	20	0.05 ± 0.06	0.02 ± 0.13			
BHVO-2¶¶	Terrestrial			ETH	20	−0.08 ± 0.03	0.01 ± 0.02	−0.01 ± 0.05	15	0.00 ± 0.07	0.04 ± 0.11			
BHVO-2	Terrestrial			ETH	19	−0.05 ± 0.05	0.04 ± 0.02	0.00 ± 0.05						
BHVO-2	Terrestrial			LLNL	6	0.03 ± 0.05	0.06 ± 0.04	−0.04 ± 0.07	20	0.02 ± 0.06	0.08 ± 0.12	10	0.02 ± 0.04	−0.02 ± 0.08
BHVO-2	Terrestrial			LLNL	9	−0.06 ± 0.13	0.05 ± 0.05	−0.01 ± 0.11				7	0.06 ± 0.05	0.05 ± 0.09
BCR-2	Terrestrial			LLNL	22	−0.04 ± 0.07	0.01 ± 0.03	−0.06 ± 0.06	12	0.03 ± 0.09	0.06 ± 0.15			
BCR-2	Terrestrial			LLNL	6	−0.02 ± 0.08	0.01 ± 0.03	−0.06 ± 0.10						
DTS-2b	Terrestrial			ETH								44	0.03 ± 0.02	0.07 ± 0.04
DTS-2b	Terrestrial			ETH								5	0.02 ± 0.04	−0.09 ± 0.10
DTS-2b	Terrestrial			ETH								14	0.00 ± 0.05	0.02 ± 0.06
DTS-2b	Terrestrial			ETH								5	0.05 ± 0.03	0.08 ± 0.07

* Powdered sample (mg).

† Dissolved sample (mg).

‡ Number of measurements.

§ Isotopic values are averages of repeats. Uncertainties are given in 2 SE (SE = SD/√*n*).

¶, #, **, ††, ‡‡, §§ Each number refers to a separate digestion; aliquots carrying the same symbol originate from the same digestion.

¶¶ Ti and Fe isotope data measured on different sample aliquots.

## Ti isotope data from (*5*).

*** Ti isotope data from (*12*).

††† Sample aliquot of (*12*), reanalyzed in this study.

**Fig. 2.**
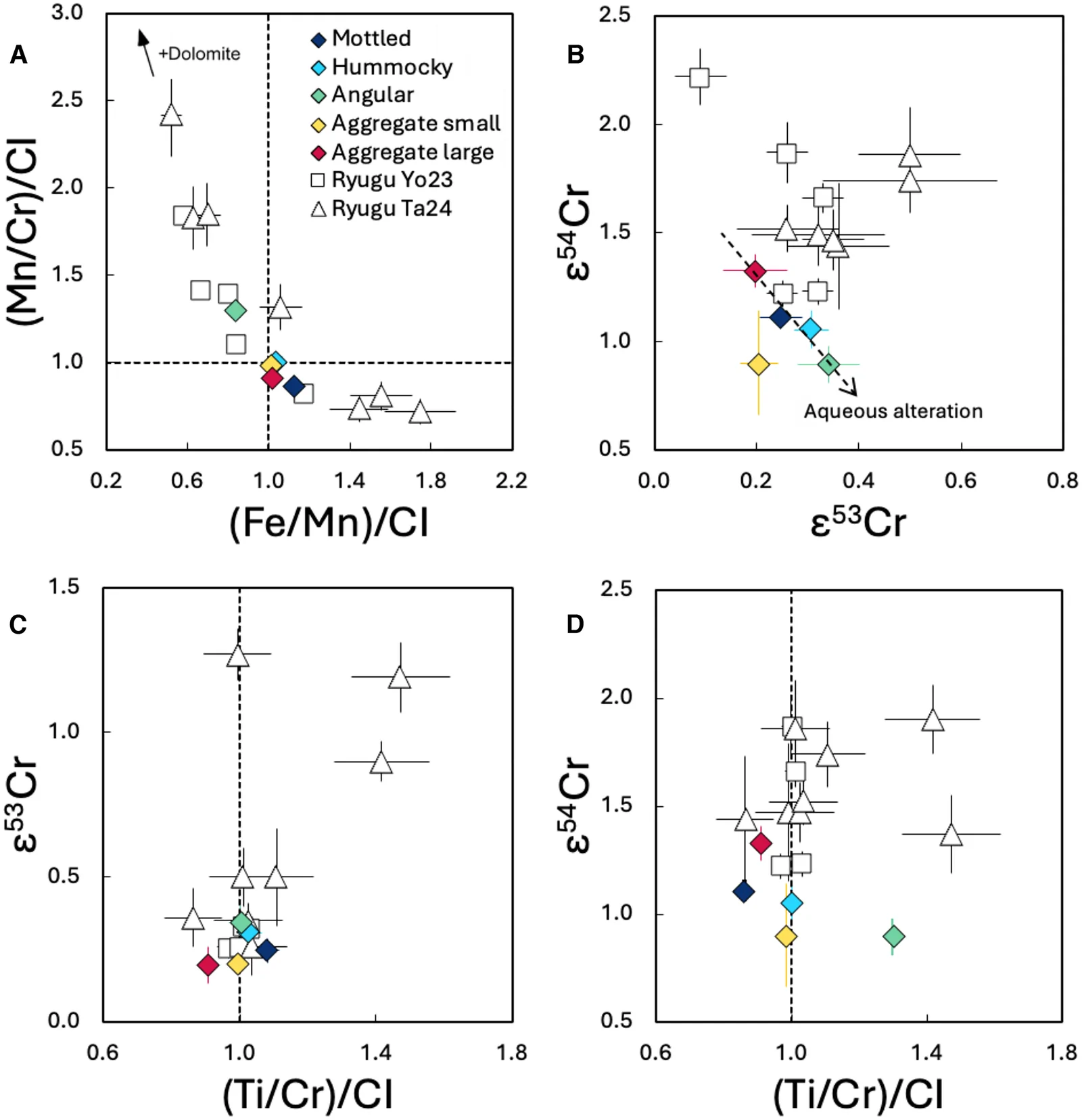
Chemical and isotopic signatures of aqueous alteration in Bennu and Ryugu. CI-normalized (**A**) Mn/Cr and Fe/Mn ratios, (**B**) ε^54^Cr and ε^53^Cr, and (**C**) ε^53^Cr and Ti/Cr ratios and (**D**) ε^54^Cr and Ti/Cr ratios for bulk samples of Bennu (colored diamonds) and Ryugu (open squares and triangles). All elemental ratios are normalized to the CI composition of (*90*). The aqueous alteration trend shown in (B) broadly follows the pattern of multiple aqueous alteration events (or the degree of fluid-rock interaction experienced by each sample) identified in (*11*), with exception of the mottled sample, which records extensive multistage alteration (*11*) but shows low dolomite content in the aliquot analyzed here (A). Bennu data are from Tables 1 to 3, and Ryugu data are from (*6*) (Yo23) and (*87*, *91*) (Ta24). The dashed lines represent the CI value from (*90*).

**Fig. 3.**
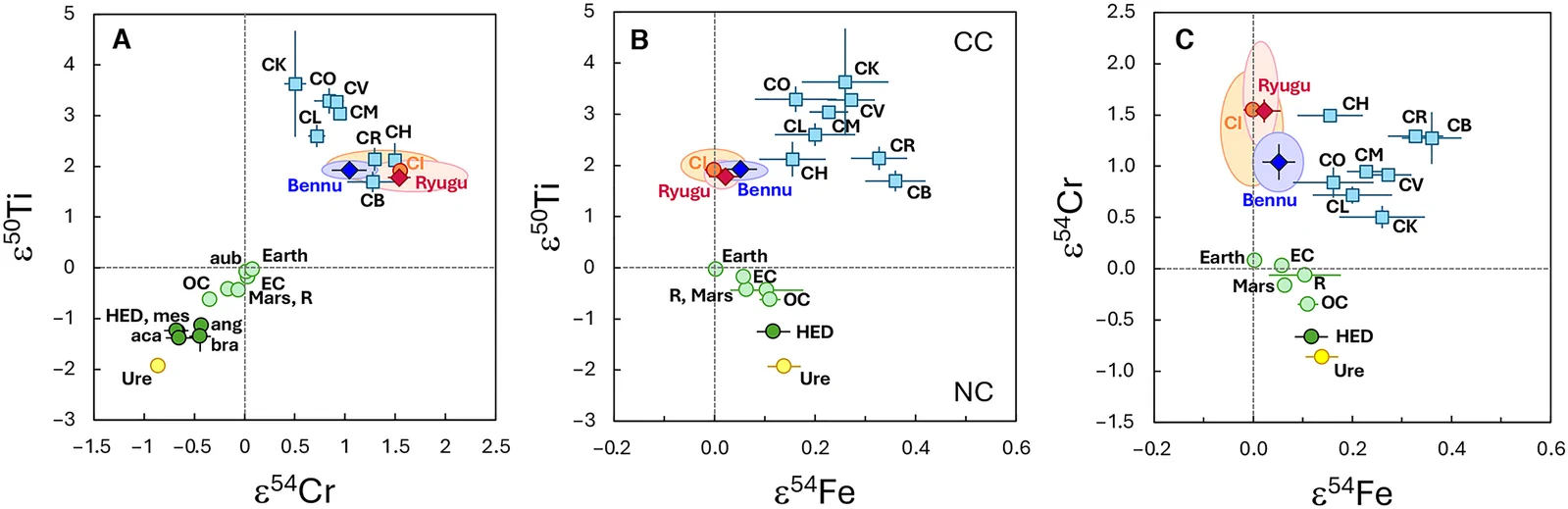
Nucleosynthetic isotope relationships of Bennu and Ryugu. Average compositions of meteorite groups shown for ε^50^Ti-ε^54^Cr (**A**), ε^50^Ti-ε^54^Fe (**B**), and ε^54^Cr-ε^54^Fe (**C**). Axes of shaded areas display the range of individual data points for Bennu (light blue), Ryugu (pink), and CI chondrites (orange). The data delineate the NC and CC reservoirs. CI chondrites and Bennu and Ryugu samples define a third reservoir in (B) and (C). The NC reservoir is divided into three subreservoirs (*12*). Shown are CCs (light blue squares and orange circle), NC bodies with three subreservoirs (light green circles for Earth-Mars region, dark green for Vesta region, and yellow for ureilites), Ryugu (red diamonds), and Bennu (dark blue diamonds). Abbreviations: aca, acapulcoites; aub, aubrites; ang, angrites; bra, brachinites; EC, enstatite chondrites; HED, howardites-eucrites-diogenites; mes, mesosiderites; OC, ordinary chondrites; R, rumuruti chondrites; Ure, ureilites. Bennu data are from Table 1 and other data from data S1 to S4. Uncertainties are 95% confidence intervals.

The ε^54^Cr data for the Bennu samples (Fig. 1C) reveal small but resolvable heterogeneity: The averages of the small and large aggregate samples are 0.85 ± 0.07 and 1.27 ± 0.09 (2 SD), respectively, whereas samples from the various lithologies span a range from the lower ε^54^Cr values (small aggregate and angular samples) to higher values (large aggregate, hummocky, and mottled). This heterogeneity is also reflected by CI samples covering an even larger range from 0.8 to 1.9 ε, with an average of 1.55 ± 0.06 (95% confidence interval) (Fig. 1C and data S1 and S4). The observed range reflects the variability among individual CI samples, whereas the average and its 95% confidence interval provide an estimate of the average ε^54^Cr composition of the CI reservoir. The large aggregate sample falls close to this average, whereas the lower ε^54^Cr values of the small aggregate and angular samples is also present in CI chondrites, although rarer in occurrence (Fig. 1C). In contrast, Ryugu data were obtained on ≤25 mg aliquots and show more scatter (from 1.2 to 2.2 ε) with a tendency to higher ε^54^Cr than Bennu (Fig. 1C). Because the ε^54^Cr data of CI chondrites cover virtually the full range identified for Bennu and Ryugu, this heterogeneity is likely inherent to CI-like material. The ε^54^Cr data of Bennu show a weak correlation with ε^53^Cr (Fig. 2B and Tables 2 and 3). The ε^53^Cr variations are dominated by radiogenic ingrowth of the short-lived ^53^Mn and were predominately produced in high Mn/Cr phases (e.g., carbonates), formed during early aqueous alteration (Supplementary Materials). This weak negative ε^53^Cr-ε^54^Cr trend supports some ^54^Cr mobility during parent body aqueous alteration. The progression we observe from higher to lower ε^54^Cr values (large aggregate > mottled ≈ hummocky ≥ angular particle types) largely follows the trend of increasing dolomite content generated by aqueous alteration (Fig. 2A and Tables 2 and 3), consistent with a low ε^54^Cr–high ε^53^Cr component as previously suggested (*6*). However, the small aggregate sample falls off the Bennu trend with low ε^54^Cr (Fig. 2B). This indicates an additional process to the formation of high Mn/Cr phases. Furthermore, other indicators of aqueous alteration, such as the correlation between ε^54^Cr and Ti/Cr ratios (tracer for magnetite + pyrrhotite), only show a weak association (Fig. 2D). This leaves room for some ^54^Cr heterogeneity reflecting initial heterogeneous distributions of the presolar carriers at a small scale, e.g., due to an uneven distribution of clasts in the different lithologies (*14*), whereas other ^54^Cr variation is linked to aqueous alteration, consistent with interpretations from Ryugu (*6*). Alternatively, all variability could be connected to aqueous alteration, e.g., involving variable degrees of nanospinel dissolution [a presolar carrier of high ^54^Cr (*15*)] and corresponding small-scale heterogeneity in the amount of ^54^Cr retained in nanospinels. Collectively, our results show that nucleosynthetic Cr isotope compositions are susceptible to small-scale modification by aqueous alteration. The observed deviations, however, are relatively small, and the link to CI chondrites remains evident (Figs. 1 and 3).

**Table 2.** Elemental concentrations and ^55^Mn/^52^Cr ratios of Bennu samples.

Sample	Lithology	^55^Mn/^52^Cr ± 2 SD	Mn/Cr ± 2 SD	Fe/Mn ± 2 SD	Ti/Cr ± 2 SD
OREX-800055-101	Angular	1.066 ± 0.023	0.944 ± 0.045	82.046 ± 2.937	0.173 ± 0.013
OREX-803231-102	Hummocky	0.823 ± 0.024	0.728 ± 0.021	101.877 ± 3.576	0.177 ± 0.010
OREX-800023-111	Mottled	0.706 ± 0.017	0.625 ± 0.019	110.611 ± 3.115	0.186 ± 0.006
OREX-803015-100	Aggregate	0.809 ± 0.014	0.716 ± 0.012	98.549 ± 1.215	0.171 ± 0.003
OREX-800117-110	Aggregate	0.746 ± 0.010	0.660 ± 0.011	99.287 ± 1.596	0.156 ± 0.008
CI chondrites			0.726	97.901	0.172

**Table 3.** Normalized elemental concentrations of Bennu samples.

Sample	Lithology, class	(Mn/Cr)/CI*,†	(Fe/Mn)/CI*,†	(Ti/Cr)/CI*,†
OREX-800055-101	Angular	1.299 ± 0.027	0.838 ± 0.018	1.003 ± 0.034
OREX-803231-102	Hummocky	1.003 ± 0.029	1.041 ± 0.012	1.026 ± 0.040
OREX-800023-111	Mottled	0.861 ± 0.020	1.130 ± 0.018	1.081 ± 0.034
OREX-803015-100	Aggregate	0.986 ± 0.017	1.007 ± 0.012	0.991 ± 0.019
OREX-800117-110	Aggregate	0.909 ± 0.013	1.014 ± 0.015	0.907 ± 0.010
CI chondrites		1	1	1

* Uncertainties are given as 2 SD.

† Normalized to the CI chondrite ratio from (*90*).

### Limited Ti and Fe isotope parent body heterogeneity in Bennu, Ryugu, and CI chondrites

Titanium and Fe isotope data reported for all five Bennu aliquots (Fig. 1 and Table 1) exhibit indistinguishable Ti and Fe isotope compositions within analytical uncertainties, revealing homogeneity in Bennu samples at the scale of 20 to 1300 mg. The mottled Bennu particle may represent an exception, exhibiting a slightly higher ε^50^Ti (2.07 ± 0.04) than the hummocky type (1.86 ± 0.08), although the difference is barely resolved within the analytical uncertainty. Mottled particles are rare; their mottled appearance is associated with late-stage evaporites, introduced along veins after formation of the phyllosilicate-rich groundmass (*2*, *9*, *11*). Their overall scarcity suggests that the heterogeneous distribution of mottled particles among bulk Bennu is unlikely to result in detectable Ti isotopic variability at current analytical uncertainties, even if these particles showed a systematic isotopic offset.

The extent of nucleosynthetic parent body heterogeneity depends on the abundance and composition of the anomalous carrier phases and their susceptibility to aqueous and thermal alteration. The ε^50^Ti trend of CCs (Fig. 3A) is typically attributed to the heterogeneous distribution of refractory inclusions with high ε^50^Ti [calcium-aluminum-rich inclusion (CAI) and amoeboid olivine aggregate (AOA) (*16*, *17*)], despite the presence of other high ε^50^Ti phases [e.g., SiC or nanospinel (*15*)] that do not substantially affect the bulk rock composition. About 45% of anhydrous minerals in Bennu yield O isotope data indicating an origin from AOAs (*18*) and thus likely carry traces of anomalous Ti. However, anhydrous minerals occur at the level of 2 to 6 wt % in the hummocky particles, whereas <2 wt % in the more aqueously altered angular particles (*18*). The identical Ti isotope data of both particle types indicate that anomalous Ti in anhydrous minerals is insufficient to affect bulk compositions despite up to threefold enrichment in hummocky particles or that Ti remained immobile during aqueous alteration and the anomalous Ti was preserved during alteration of anhydrous minerals to phyllosilicates. The Ti and Fe isotope homogeneity of Bennu samples highlight their robustness to small-scale aqueous alteration, whereas Cr isotopes retain evidence of minor modification. Moreover, the higher abundance of clastic inclusions in hummocky particles (*14*) has no measurable effect on the bulk Ti and Fe isotope compositions.

The Ti and Fe isotope data of all Bennu samples overlap with those of Ryugu and CI chondrites within analytical uncertainty (Fig. 1), demonstrating broad homogeneity on a >20 mg level for CI-like bodies, despite minor Ti isotope variations previously reported among Ryugu particles (≤25 mg) (Fig. 1A). Thus, provided that sufficiently large sample masses are analyzed, nucleosynthetic Ti and Fe isotope compositions preserve the distinct isotopic fingerprint of the primary dust incorporated into parent bodies. This conclusion is reinforced by the additional broad Cr isotope homogeneity of Bennu, Ryugu, and CI chondrites, which show that nucleosynthetic compositions are only minimally overprinted by subsequent processes, including parent-body aqueous alteration or the disruption and reaccretion experienced by Bennu before becoming a near-Earth rubble-pile asteroid (*4*).

### Shared accreted dust reservoirs for Bennu, Ryugu, and CI chondrites

**Fig. 4.**
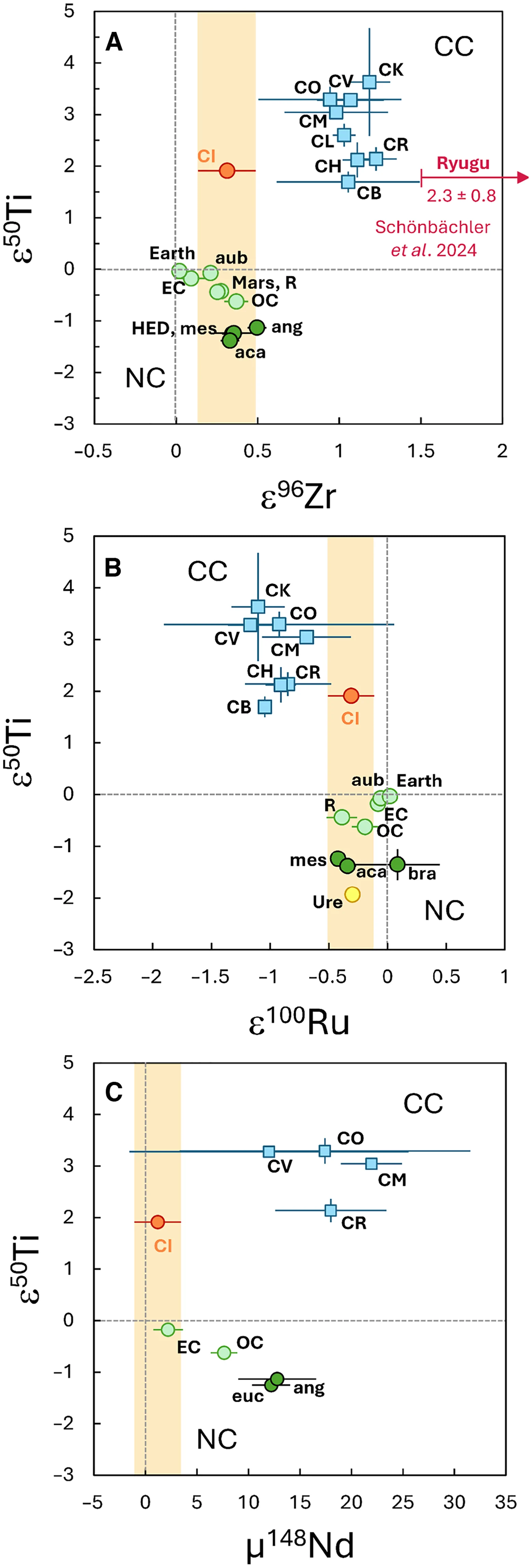
Nucleosynthetic isotope reservoirs in the Solar System. Average compositions of meteorite groups for ε^50^Ti versus ε^96^Zr (**A**), ε^100^Ru (**B**), and μ^148^Nd (**C**). The data define the NC and CC reservoirs. CI chondrites define a third reservoir. The Zr isotope composition of Ryugu most likely reflects sample heterogeneities (*92*) due to a depletion of SiC grains in the analyzed material, potentially caused either by incomplete sample digestion or by true variability in the sample. Shown are CCs (light blue squares and orange circle; orange shaded areas represent the average and 95% confidence intervals of CI) and NC bodies with three subreservoirs (light green circles for Earth-Mars region, dark green for Vesta region, and yellow for ureilites). Abbreviations: aca, acapulcoites; ang, angrites; aub, aubrites; bra, brachinites; EC, enstatite chondrites; HED, howardites-eucrites-diogenites; euc, eucrites; mes, mesosiderites; OC, ordinary chondrites; R, rumuruti chondrites; Ure, ureilites. Data sources are provided in data S1 to S6.

The overlapping Ti, Cr, and Fe isotope data corroborate a close genetic relationship between samples of Bennu, Ryugu, and CI chondrites (Figs. 1 and 3). Therefore, Bennu, Ryugu, and the CI chondrite parent bodies incorporated a similar assemblage of nucleosynthetic material, suggesting that they formed from similar dust reservoirs in the same region of the protoplanetary disk or they may even trace back to a single primordial parent body. Although the CI-like material plot together with the CR clan (CR, CB, and CH chondrites) in ε^54^Cr-ε^50^Ti space (Fig. 3A), ε^54^Fe data uniquely set them apart from all other CC groups including the CR clan (Fig. 3, B and C). Their ε^54^Fe values overlap with those of Earth and enstatite chondrites, indicating a closer link to the noncarbonaceous chondrite (NC) reservoir of the inner Solar System than other bodies from the CC reservoir (Fig. 3). This affinity to the NC reservoir is further reinforced by recent ε^96^Zr data for CI chondrites (*19*), which—similar to ε^54^Fe—are distinct from other CCs and overlap with NC compositions (Fig. 4A). A comparable overlap between CI-like materials and the NC field has also recently been identified for Nd (*20*) and Ru isotopes (*21*) (Fig. 4, B and C). These converging observations underscore the unique composition of CI-like bodies and their unusually close genetic relationship to NC materials compared with other CC groups.

## DISCUSSION

### Where did Bennu’s parent asteroid and other CI-like bodies accrete?

The nucleosynthetic isotope compositions of multiple elements demonstrate that Bennu and other CI-like bodies are unlikely to be derived from either the NC or CC reservoirs alone. Instead, they appear to record a distinct feeding zone with isotopic affinities to both reservoirs. This observation provides a key constraint on the formation environment of CI-like bodies, requiring a mechanism capable of generating and preserving such an intermediate fine-grained reservoir.

The region from which Bennu’s parent body accreted was populated by a diverse suite of solids sourced from a wide range of environments and heliocentric distances, implying that the CI-formation region broadly sampled many parts of the protoplanetary disk. This included anhydrous minerals, presolar grains, along with organic matter inherited from the molecular cloud and the cold outer regions of the disk, all of which were incorporated into Bennu (*2*, *5*, *14*). Among these components, the volatile inventory provides a strong constraint on the formation environment. Bennu samples record incorporation of ices dominated by H_2_O, and this places the accretion region of their parent body outside the water ice line. Accreted ices also include CO-, CO_2_-, NH_3_-, and other N-bearing species (*22*, *23*), indicating that formation beyond the NH_3_ ice line is plausible, although not strictly required. This is consistent with evidence from the Rosetta mission, showing that other volatiles can be preserved in water ice (*24*), thereby raising the temperatures at which they sublimate. Together, the volatile and nucleosynthetic signatures strongly suggest that Bennu’s parent body belongs to the CC reservoir (Fig. 3) and support its formation in a cold distal region of the protoplanetary disk beyond the water ice line.

Both Bennu and Ryugu are now near-Earth rubble-pile asteroids, and they must have migrated inward from the formation region of their parent asteroid(s) (*4*). On the basis of nucleosynthetic Fe isotope data, Hopp *et al.* (*25*) proposed that Ryugu’s parent asteroid may have formed far out, beyond the formation region of Jupiter or Saturn, distinct from other CCs (e.g., CV, CO, and CM), and was later transported to the inner Solar System through dynamical excitation by Uranus and Neptune. This model suggest that CI-like bodies may have accreted in the same region as comets, which presents challenges. First, CI materials do not exhibit the high ^15^N and deuterium enrichments commonly associated with cometary matter (*1*, *26*–*28*), although some of these signatures might have been muted by thermal and parent body processing (*29*). Second, the occurrence of a dark clast in a CR chondrite—whose chemical composition, organic-rich petrology and ^15^N- and D-rich, ^16^O-poor signatures are consistent with an outer-disk, likely cometary origin (*30*)—is consistent with Mg and Cr isotope data for such clasts (*27*). Together with the distinct bulk nucleosynthetic composition of CR chondrites (Fig. 3, B and C), these observations suggest that the CR parent body sampled material from a reservoir located closer to the comet-forming region than that sampled by CI chondrites, although alternative interpretations remain possible. Third, new chronometric constraints point to early formation of CI bodies. Although CI chondrite parent body ages of 3.6 ± 0.5 million years (Myr) after CAI formation were previously proposed (*31*), recent Ryugu data (*32*) indicate formation as early as ∼2 Myr. Our Bennu Mn-Cr systematics also allow for such early formation (Materials and Methods). Thus, CI bodies appear to have formed earlier than CV, CO, and CM chondrites [3 to 4 Myr (*31*)] and substantially earlier than CR chondrites [3.7 to 4.5 Myr (*33*)]. This timing is difficult to reconcile with a formation in the comet forming region and the idea that low surface densities in the outer disk delayed comet and Kuiper Belt object formation until late stages of disk dispersal (*34*, *35*). It also conflicts with recent formation scenarios in which planetesimals first preferentially formed from coarser dust, such as chondrules and refractory inclusions (e.g., represented in CV and CO chondrites) and only after these particles were depleted, finer-grained bodies could form (e.g., CM and CI chondrites) (*36*, *37*). This predicts a relatively late formation of CI bodies. Accepting an early accretion of CI-like bodies therefore requires an alternative explanation. Such a scenario must not only account for the emerging early accretion age of CI-like bodies but also explain their distinctive nucleosynthetic isotope signatures. The isotope data (Figs. 2 and 3) require a formation environment capable of producing a dust reservoir with isotopic affinities to both the NC and CC reservoirs. Although such a reservoir could, in principle, have existed farther out within the carbonaceous domain, formation near the water ice line provides the most natural physical setting for generating it through inward transport of fine-grained dust while Jupiter simultaneously filtered larger particles. We therefore evaluate the alternative scenario in which Bennu and other CI-like parent bodies formed just outside the water ice line, interior to Jupiter’s orbit, at ∼2 Myr after CAI formation; a similar scenario was independently developed by van Kooten *et al*. (*38*).

### Disk structure and size-selective transport imposed by Jupiter

Jupiter likely formed early, within the first million years after CAI formation (*39*–*41*), potentially accreting its initial core from planetesimals that formed at the water ice line (*39*, *42*). In such models, Jupiter has grown to >20 Earth masses at 2 Myr (*39*). A protoplanet of this mass would have opened a gap in the gas disk and generated pressure maxima capable of initiating planetesimal formation both interior and exterior to Jupiter’s orbit (*43*). In addition, giant planets act as size-selective transport barriers (*43*–*46*). Small, gas-coupled solids and fragments can move through Jupiter’s gap into the inner Solar System, whereas larger, less strongly coupled particles become increasingly trapped in the pressure maxima outside Jupiter. The effective filtering size is not characterized by a single particle diameter, but depends on specific disk conditions (*47*) and shifts to smaller sizes as Jupiter grows (*43*). Observations of protoplanetary disks show ring structures dominated by approximately millimeter-sized particles together with evidence for finer material persisting within disk gaps (*48*, *49*). Collisional fragmentation continually regenerates these small grains, which remain gas-coupled and can drift inward through Jupiter’s gap (*45*, *46*). As a result of this evolving disk, only a subset of solids dominated by small grains and fragments sampling multiple source regions was transported inward across Jupiter’s orbit and made available for accretion by CI-like parent bodies. The same transport process also supplied some refractory material to planetesimals forming in the inner Solar System.

### Nucleosynthetic fingerprints of size filtering and radial drift

The effects of this size-selective filtering are recorded in nucleosynthetic isotope compositions. High ^50^Ti abundances are primarily carried by CAIs and AOAs (*16*, *17*). The abundances of these inclusions decrease from CV to CM chondrites, reaching low levels in CI-like and inner Solar System materials such as ordinary chondrites (*50*, *51*), whereas their size distributions tends to be dominated by smaller inclusions and fragments. The corresponding ε^50^Ti values (Fig. 3A) mirror the abundance trend and mass balance considerations indicate that variations in CAI abundances can account for much of the observed variations among CCs (*16*), including the ε^54^Cr-ε^50^Ti trend (*52*). However, the jump to very low ε^50^Ti values in the inner Solar System (−1.93 ± 0.07 for ureilites; data S1) requires an additional process beyond CAI filtering that lowers Ti isotope ratios. Possible mechanisms include the local addition of molecular-cloud material with distinct supernova signatures (*27*, *53*, *54*) or preferential loss of a ^50^Ti-rich carrier if the Ti isotopic signature was preferentially hosted in ices (*19*). Jupiter-induced size filtering of CAIs can further explain refractory element variations across the Solar System (*40*). Ordinary chondrites generally contain small CAIs [<100 to 200 μm (*47*, *50*)], indicating that material larger than this was mostly prevented from crossing Jupiter’s gap.

A similar argument applies to chondrules. The very low abundance of intact chondrules in CI materials is consistent with Jupiter inhibiting inward transport of millimeter-scale chondrules, whose typical sizes in CCs range from ∼0.15 to 1 mm (*55*). Oxygen isotope data of olivine grains in Bennu samples indicate that they originate from both AOAs and chondrules [45:55% (*18*)], in line with results from Ryugu and the Ivuna CI chondrite (*56*). Thus, chondrule fragments crossed the gap and were incorporated in Bennu’s parent asteroid. This requires that chondrule formation in the CC reservoir was underway by 2 Myr, consistent with chronological constraints (*57*). In contrast, chondrules in ordinary chondrites exhibit O isotope characteristics of the NC reservoir and are generally interpreted to have formed in the inner disk. However, a subset of smaller chondrules (<300 μm) is potentially of CC reservoir origin, consistent with Jupiter as a size-selective filter (*58*).

In summary, the size-selective filtering of larger nucleosynthetic carriers (e.g., intact CAI, AOA, and chondrules) by Jupiter likely contributed to first-order isotopic differences between the NC and CC reservoir, particularly the enrichment of supernova-derived isotopes (e.g., ^50^Ti) in the CC reservoir (*12*). More generally, however, the nucleosynthetic distinction between the NC and CC reservoirs extends well beyond the isotope variations discussed here and is considerably more complex than implied by any single isotope signature or stellar-source interpretation (*57*, *59*). In addition, part of the observed isotopic differences may reflect the late incorporation of localized molecular cloud material with contrasting abundances of supernova-derived isotopes, either supernova-depleted in the inner disk (*54*) or supernova-enriched in the outer disk (*27*). The distinct yet coherent isotopic sequence among NC bodies, from Earth-like to Vesta-like materials and ureilites (Figs. 3 and 4), and their correlated trends across many elements are consistent with progressive thermal processing in the inner disk (*19*, *53*). By contrast, the opposite behavior of Ti and Cr isotopes in the CC reservoir (with CI materials showing lowest ^50^Ti but highest ^54^Cr values) is well explained by size sorting and the variable accretion of different dust components, including CAIs.

### Parent body formation and water contents

Accretion of CI-like materials just beyond the water ice line, interior to Jupiter’s orbit is consistent with planetesimal formation by streaming instabilities, a leading mechanism for rapid assembly of small bodies. The water ice line is the preferred location for triggering streaming instability (*60*). At the water ice line, sticking properties between dry and icy particles change thereby creating a “traffic jam” that slows inward drift (*60*). Evaporation and outward diffusion of water vapor result in recondensation just outside the ice line, increasing the dust-to-gas ratio and triggering planetesimal formation. During these processes, ice rims grow on existing particles, increasing their sizes and water content, and naturally yield the relatively high water abundances [6 to 20 wt % (*2*, *3*)] observed in Bennu, Ryugu, and CI chondrites compared with other chondrite groups. In contrast, other less water-rich CCs may have formed in pressure maxima outside Jupiter’s orbit induced by the giant planets. This scenario remains consistent with water ice accretion by NC bodies, including those of ordinary and R chondrites (*61*). At the relevant time, the inward migration of the water ice line allows water ice to be incorporated inside the formation region proposed for CI bodies, naturally accounting for both their modest water inventories and their formation ages, which coincide with that of Ryugu (*32*).

**Fig. 5.**
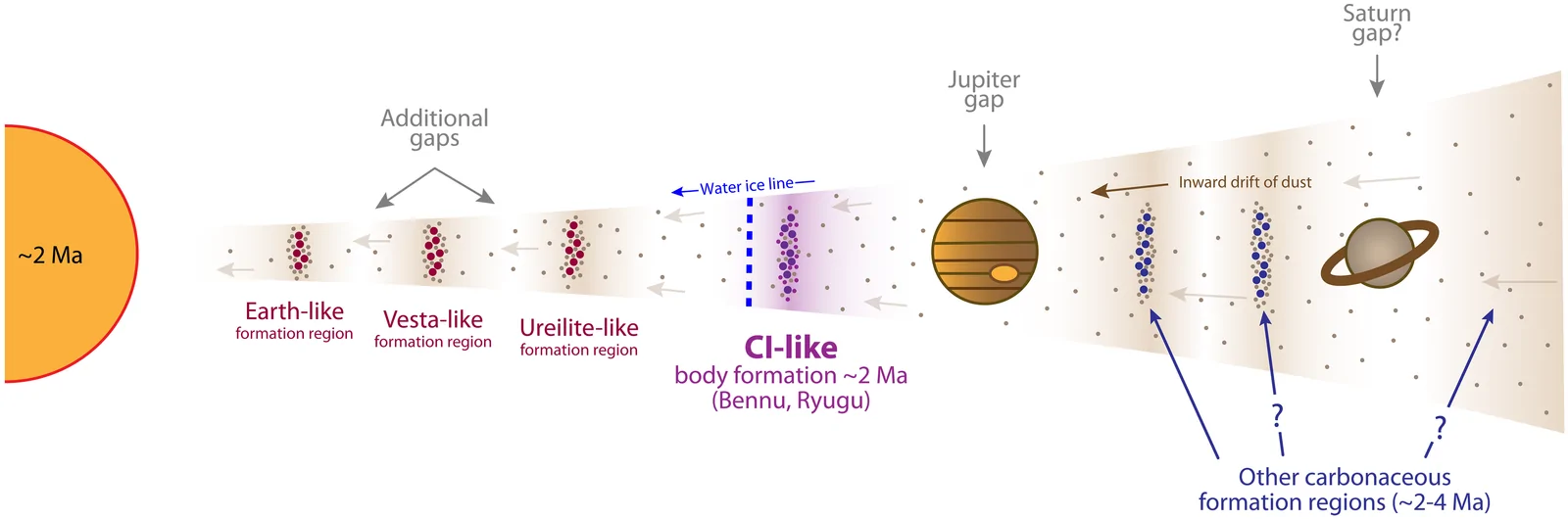
Dust rings in the solar protoplanetary disk. The water ice line generates a pressure maximum just beyond the sublimation front, promoting dust growth by condensation, followed by dust trapping and planetesimal formation via streaming instabilities; we propose this region as the preferred formation site of CI-like parent bodies. In contrast, fine-grained particles remain well coupled to the gas and are largely unaffected by pressure bumps, allowing them to drift across it. Gaps in the inner Solar System (inside the ice line; NC reservoir) may have been produced by spiral density waves associated with Jupiter’s growth, with Saturn contributing to additional structure, particularly exterior to Jupiter’s orbit. Gaps in the NC reservoir are motivated by the identification of three isotopic NC subreservoirs (Fig. 2) (*12*). Pressure maxima within the rings enhance dust concentrations and facilitate planetesimal formation, leading to distinct subreservoirs with characteristic isotopic signatures. These reservoirs are reflected in CI-like, Ureilite-like, Vesta-like, and Earth-Mars–like clusters in Ti, Cr, and Fe isotope space (Fig. 3). The inner and outer Solar System reservoirs differ in numerous nucleosynthetic isotope systems and volatile abundances. Here, we highlight the contrast in volatile contents and selected supernova-derived isotope signatures (e.g., ^50^Ti and ^54^Cr), with the NC reservoir being depleted and the CC reservoir enriched in these isotopic components. Ma, million years.

By forming at the water ice line inside Jupiter (Fig. 5), a transitional zone between the inner and outer disk (*42*), CI-like bodies sampled a unique dust inventory. This explains their dual nature because they are physically and chemically carbonaceous due to their origin just beyond the ice line while exhibiting isotopic compositions that fall between those of the NC and CC reservoirs (Figs. 3 and 4). This transitional isotopic character suggests they accreted from a distinct fine-grained reservoir that was less influenced by some of the distally formed CC components (e.g., intact CAIs and chondrules) compared to other CCs due to size-selective filtering of Jupiter.

### Why do Bennu, Ryugu, and CI chondrites mirror solar composition in many aspects?

Among all known astromaterials, only samples from Bennu, Ryugu, and CI chondrites exhibit bulk elemental compositions that closely mirror the solar photosphere (*2*, *6*). Petrographic observations of these samples show that their fine-grained, phyllosilicate-dominated matrix is largely secondary, reflecting extensive parent body aqueous alteration and brecciation (*11*, *14*). However, the samples still contain small amounts of surviving anhydrous silicates (typically 2 to 6 wt %), interpreted to represent fragments of chondrules and refractory inclusions (*18*). In contrast, cosmochemical observations of primitive, virtually unaltered chondrite matrix indicate primitive dust consisting of intimately mixed fine-grained components [typically <5 μm (*62*)] of diverse composition such as amorphous silicates, organic matter, presolar grains, and fragments of anhydrous silicates (*63*, *64*).

Recent disk models that include growing giant planets indicate that such fine-grained material can be efficiently mixed across large radial distances by diffusion, virtually unaffected by gaps and pressure maxima (*45*, *65*). Moreover, particle growth and fragmentation driven by collisions occur both within the disk and during planetesimal impacts, releasing small dust particles (*46*). This continual grinding of larger solids also added material to the fine-grained fraction. Because these fine particles remain tightly coupled to the gas, turbulent diffusion and radial transport redistribute them across large regions of the disk on timescales much shorter than planetesimal formation (*45*, *65*). Consequently, CI-like bodies sampled the dominant fine-grained background dust reservoir of the disk. The coexistence of a near-solar bulk composition with surviving anhydrous silicates in Bennu demonstrates that fragments of thermally processed material were incorporated into this reservoir, but only as a minor component.

In this framework, the chemically well-mixed fine-grained dust reservoir largely controlled the bulk elemental compositions of CI-like bodies, whereas other chondrite groups incorporated variable proportions of this background reservoir together with locally concentrated coarse components, such as chondrules and refractory inclusions (*66*, *67*), leading to systematic deviations from solar composition. Whether the chemically well-mixed component itself was uniform across all regions of the disk remains debated (*63*), and it may have been modified locally by chondrule formation and related processes (*68*). Jupiter’s early growth may have further influenced this reservoir by limiting the inward transport of larger solids such as intact CAIs and millimeter-scale chondrules while allowing smaller fragments and gas-coupled grains to pass (*47*). Consistent with this, high-temperature components in Bennu appear predominantly as fragments, suggesting extensive preaccretional processing (*18*).

The near-solar compositions of CI-like materials may thus emerge from sampling a compositionally diverse population of fine-grained solids, enabled by efficient mixing of fine-grained material. This is analogous to fine-grained loess or shale sediments, which are widely regarded as compositional averages of heterogeneous continental crust derived from continental debris. Whether CI-like bodies also reflect the average solar nucleosynthetic composition remains uncertain as isotopic and chemical signatures can be readily decoupled. Many strongly anomalous isotopic carriers do not significantly contribute to the bulk elemental compositions, allowing materials to appear chemically “solar” while remaining isotopically distinct. Ultimately, our results suggest that the “solar” nature of CI-like bodies is a direct consequence of their formation at the water ice line inside Jupiter’s orbit. This location allowed them to accrete from an inward-drifting, well-mixed reservoir while remaining far enough out to retain the volatile-rich, carbonaceous character of the outer Solar System.

## MATERIALS AND METHODS

### Sample preparation

All samples were homogenized into fine powders using a high-purity quartz mortar and pestle before splitting and taking aliquots. In between samples, the mortar and pestle were cleaned several times by grounding high-purity quartz sand with 1 M HCl, isopropanol, and Milli-Q water. The samples were analyzed at ETH Zurich and at the Lawrence Livermore National Laboratory (LLNL) (Table 1). All aliquots were processed through a succession of extraction and ion-exchange chromatography columns to separate Ti, Fe, and Cr from the sample matrix for isotopic analysis with a Thermo Fisher Scientific Neptune Plus MC-ICP-MS or Thermo Fisher Scientific Neoma.

### Sample dissolution

All samples labeled with LLNL in Table 1, Aguas Zarcas, and all OREX samples (except OREX-800117-110 and OREX-803015-100/-101) were dissolved at the LLNL. The powdered samples were dissolved in Savillex PFA beakers with a 2:1 mixture of concentrated HF and HNO_3_ at 180°C for at least 4 days. These solutions were dried, and residues were redissolved in reverse aqua regia at 140°C for at least 3 days. Solutions were dried again and redissolved in 6 M HCl. If any residual solids were visible at this step, these were centrifuged and transferred into a Parr bomb and dissolved in 2:1 HF and HNO_3_ in an oven at 200°C for 48 hours. Once fully dissolved, all samples were converted to 6 M HCl.

Sample OREX-803015-100/-101 was dissolved at Washington University, St. Louis (*5*). First, 1 ml of H_2_O_2_ was added to the powder, followed by concentrated HNO_3_-HF. The sample was left on a hotplate at 180°C for ∼3 days, then dried down, and redissolved in concentrated HCl-HNO_3_ (2:1) on a hotplate at 180°C for ∼2 days. The sample was dried down and redissolved twice in 6 M HCl.

The ETH aliquot of the large homogenized aggregate powder (OREX-800117-110) was obtained as a powder slurry in ultrapure water. The sample was then split into three vials, adjusted to achieve an even distribution of sample powder in each vial, and then dried down at 40°C. After evaporation, 1 ml of concentrated HF and 0.3 ml of concentrated HNO_3_ were added to each vial. The vials were placed into Parr bombs and left in an oven for 5 days at 170°C following the procedure of (*69*), which was also used to digest Ivuna PB. The samples were then evaporated to dryness before dissolution in 10 ml of concentrated HNO_3_. The solutions were dried down again and dissolved in 10 ml of 6 M HCl after which the sample was fully dissolved. The three solutions of OREX-800117-110 were then combined. A 3.7% aliquot (17.86 mg) was used for Ti, Fe, and Cr isotope analyses at ETH Zurich. All other samples dissolved at ETH (listed in Table 1 under ETH, except the OREX samples and Aguas Zarcas) were first dissolved in 1.5 ml of concentrated HNO_3_ and 1.5 ml of concentrated HF in square Savillex vials and heated in an oven at 155°C for 3 days. After drying down, the residues were subsequently redissolved in 3 ml of concentrated HCl and left on a hotplate at 150°C for 3 days. Following this, the solutions were again dried and redissolved in 6 M HCl. This final dissolution step was repeated until the solutions were completely clear.

### Chemical separation procedure and isotope analyses at ETH Zurich

Titanium was separated from the sample matrix using a three-stage anion-exchange chromatography using AG1-X8 resin (*69*, *70*). Samples were loaded in 4 M HF for the first and the third columns, and Ti was eluted in 6 M HCl–1 M HF. The main matrix fraction eluted in 4 M HF of the first separation stage was combined with potential fluorides formed and centrifuged before sample loading. This combined fraction is referred to as the “Ti matrix fraction” further below and was processed further to isolate Fe and Cr. Titanium was separated from Zr and Hf in the second separation step where samples were loaded and Ti is eluted in 0.25 M H_2_SO_4_–1% H_2_O_2_. Total Ti procedural blanks ranged from 1 to 3 ng of Ti corresponding to a negligible blank contribution of <0.1%.

For Cr separation, the Ti matrix fraction was used and this fraction contained ∼100% of the Fe and Cr of the sample. Typically, 70 to 80% of this fraction underwent the Cr purification procedure, with the remaining material reserved for Fe purification. Chromium was separated from the matrix and from major isobaric interferences (Fe, Ti, and V) using a two-stage ion-exchange procedure with AG50-X8 cation resin, as described in (*71*), adapted from (*72*, *73*).

The first column used 4 ml of AG50-X8 resin in Savillex PFA columns. Before loading, samples were refluxed in 0.4 ml of 9.6 M HCl for at least 24 hours at 110°C to ensure reduction of Cr to Cr^3+^. Approximately 20 min before loading, the sample was removed from the hotplate and diluted to 1 M HCl. Chromium was collected from the loading fraction and a subsequent 14-ml step of 1 M HCl. Other matrix elements were then eluted in 14 ml of 6 M HCl. The Cr fraction from the first column was taken to dryness and refluxed in 0.2 ml of 1 M HNO_3_ for 24 hours. Once cooled to room temperature, 0.02 ml of 30% H_2_O_2_ was added, and the solution was left to equilibrate for at least 4 days to fully oxidize Cr to Cr^6+^. This was then loaded onto polypropylene columns containing 0.4 ml of AG50-X8 resin. After loading, Ti, V, and other matrix elements were eluted in 10.2 ml of 1 M HNO_3_, followed by Cr elution in 2.4 ml of 6 M HCl. The final Cr fraction yielded >95% recovery. The procedural blank for the entire ion chromatography procedure was negligible (<100 ng of Cr) compared to the amount of Cr purified (>10 μg).

For Fe purification, 20 to 30% of the Ti matrix fraction was processed. Iron was separated from isobaric interferences (Ti and Cr) and other matrix elements using one of two only slightly different, ion-exchange protocols. Both protocols used a single stage using polypropylene columns loaded with 1 ml of AG1-X8 anion resin. The first protocol, based on (*74*, *75*), involved dissolving the sample in 0.4 ml of 6 M HCl and refluxing overnight at 80°C before loading. After loading, matrix elements were eluted in 16 ml of 6 M HCl, followed by Fe elution in 5 ml of 0.4 M HCl. The second protocol, based on (*76*), used 0.4 ml of 9 M HCl to dissolve the sample. After loading, an additional 2.5 ml of 9 M HCl and 16 ml of 6 M HCl were added to remove matrix elements. Iron was then eluted in 15 ml of 0.4 M HCl. Both protocols achieved near-quantitative Fe yields. The procedural blank was negligible (<13 ng of Fe) compared to the typical amount of purified Fe (1000 μg).

Isotopic compositions for Ti were determined on a Thermo Fisher Scientific Neptune Plus MC-ICPMS using X skimmer and standard sample cones in medium resolution (6500 < *M*/Δ*M* < 7500) on the flat low-mass peak shoulder in combination with an Aridus II desolvating nebulizer system for sample introduction (*12*). The sample and standard solutions were introduced in 0.5 M HNO_3_–0.015 M HF. Titanium signals on ^48^Ti ranged from 27 to 44 V on a 10^11^-Ω amplifier for Ti solutions containing 340 to 670 ng/g Ti. Analyte Ti content varied depending on daily sensitivity and was matched for samples and bracketing standards. Sample and standard measurements were analyzed dynamically including 40 integrations of 8.4 s (^44^Ca and all Ti isotopes) and 4.2 s (^49^Ti, ^51^V, and ^53^Cr). All ion beam intensities were corrected using on-peak background analyses. Interferences of Ca, V, and Cr were corrected using measurements of ^44^Ca, ^51^V, and ^53^Cr. Titanium isotope ratios were internally normalized to a ^49^Ti/^47^Ti ratio of 0.749766 (*77*). Results are reported using standard sample bracketing relative to an Alfa Aesar Ti wire standard as: ε*^x^*Ti = [(*^x^*Ti/^47^Ti)__sample__/(*^*^x^*^*Ti/^47^Ti)_Alfa Aesar Ti wire standard_ − 1] × 10,000, for *x* = 46, 48, and 50 (*12*). An outlier correction based on 2 SD is applied to reported average results. External reproducibility (2 SD) based on BHVO-2 over 8 months is ±0.19 for ε^46^Ti, ±0.10 for ε^48^Ti, and ±0.18 for ε^50^Ti.

Chromium isotope measurements were performed on a Thermo Fisher Scientific Neptune Plus MC-ICPMS equipped with a Cetac Aridus II desolvating nebulizer system. Analyses were conducted in medium-resolution mode and using an H skimmer and standard sample cone. The optimal measurement position on the interference-free shoulder was established at the start of each analytical session through a series of measurements across the shoulder plateau. The ^52^Cr signal was measured using a 10^10^-Ω amplifier, whereas the remaining Cr isotopes were collected on 10^11^-Ω amplifiers. Each sample and standard measurement consisted of 100 integrations of 4.2 s. The preceding on-peak background measurement consisted of 50 integrations of 4.2 s and was used for a background correction. Isobaric interferences were monitored simultaneously by measuring ^49^Ti and ^56^Fe on 10^12^-Ω amplifiers and ^51^V on a 10^11^-Ω amplifier. Sample and standard solutions were diluted to 4 to 5 μg/g Cr in 0.5 M HNO_3_, producing ^52^Cr signals of ∼110 V.

Sample analyses were bracketed by two NIST SRM 979 Cr standards, matched to within 10% of the sample concentration. Sample and standard data were corrected for instrumental fractionation using the exponential law and assuming a ^50/52^Cr ratio of 0.051859 (*78*). Sample data are expressed as: ε*^x^*Cr = [(*^x^*Cr/^52^Cr)_sample_/(*^x^*Cr/^52^Cr)_NIST SRM 979 Cr standard_ − 1] × 10,000, for *x* = 53 and 54 relative to the NIST SRM 979 Cr bracketing standards. The terrestrial standard DTS-2b was analyzed multiple times in each analytical session to monitor daily accuracy and long-term reproducibility. The long-term reproducibility based on multiple aliquots of DTS-2b measured during this project is ε^53^Cr = 0.03 ± 0.14 and ε^54^Cr = 0.05 ± 0.25 (*n* = 68; 2 SD).

Iron isotope analyses were performed on a Thermo Fisher Scientific Neptune Plus MC-ICPMS using an H skimmer and standard sample cone. The sample was introduced via a Scott double-pass quartz cyclonic spray chamber and a nebulizer (∼100 μl/min). Measurements were acquired in medium-resolution mode, on the flat-topped plateau of the low-mass shoulder of each peak. The optimal measurement position was established at the start of each analytical session by a series of measurements across the shoulder plateau. The ^56^Fe isotope was measured using a 10^10^-Ω amplifier, whereas the remaining Fe isotopes were collected on 10^11^-Ω amplifiers. Isobaric interferences from Cr and Ni were monitored simultaneously via ^53^Cr and ^60^Ni, both measured on 10^12^-Ω amplifiers. Sample and standard solutions were diluted to Fe concentrations of 20 to 25 μg/g in 0.42 M HNO_3_, yielding a typical ^56^Fe signal intensity of 240 V. Each measurement was preceded by a background measurement consisting of 10 integrations of 8.4 s each, but no background correction was applied. Sample and standard analyses comprised 20 integrations of 8.4 s. Each sample was bracketed by two IRMM-524a Fe standards, matched to within 10% of the sample concentration. Samples were typically analyzed in blocks of five analyses of the same solution, with standards interwoven throughout. Instrumental mass fractionation was corrected using the exponential law, applying multiple normalization schemes: ^57^Fe/^56^Fe = 0.023096, ^57^Fe/^54^Fe = 0.36255, ^58^Fe/^57^Fe = 0.133028, ^58^Fe/^56^Fe = 0.003072, and ^54^Fe/^56^Fe = 0.063703 (*79*) to assess data accuracy. The sample data are reported in epsilon relative to the IRMM-524a Fe bracketing standards using the ^57^Fe/^56^Fe normalization as: ε*^x^*Fe = [(*^x^*Fe/^56^Fe)_sample_/(*^x^*Fe/^56^Fe)_IRMM-524a Fe Standard_ − 1] × 10,000, for *x* = 54 and 58.

The terrestrial reference sample BHVO-2 was analyzed in each session to monitor daily accuracy and to assess long-term reproducibility. The long-term reproducibility of multiple aliquots of BHVO-2 measured during this project, for the ^57^Fe/^56^Fe normalization scheme, is ε^54^Fe = 0.03 ± 0.26 and ε^58^Fe = −0.01 ± 0.48 (*n* = 50; 2 SD).

### Chemical separation procedure and isotope analyses at the LLNL

Iron, Ti, and Cr were separated from one another and from the sample matrix following the methods outlined in (*80*), combining ion-exchange chemistry procedures previously established at the LLNL (*81*–*83*).

First, Fe was separated using 2 ml of Bio-Rad AG-1X8 (100 to 200 mesh) resin in Bio-Rad Poly-Prep columns (*83*). After cleaning and conditioning the resin, samples were loaded in 2 ml of 7 M HCl–0.01% H_2_O_2_ and the columns rinsed with another 9 ml of the same solution. At this molarity, Fe^3+^ sticks to the resin, whereas Ti, Cr, Ni, and along with most other elements are collected for further purification. Iron was then collected with 9 ml of 0.5 M HCl–0.5 M HF. Last, Zn (along with Mo) was collected with 9 ml of 3 M HNO_3_. This column was repeated once to ensure purified Fe fractions. Next, Ti was purified using 2 ml of DGA resin cartridges from Eichrom in combination with an Eichrom vacuum box, slightly modified from (*84*). After precleaning and conditioning, samples were loaded in 2 ml of 12 M HNO_3_. Matrix elements (Ca, Cr, Ni, etc.) were collected with an additional 10 ml of 12 M HNO_3_ before Ti was collected with 12 ml of 3 M HNO_3_. Ti was further purified using 2 ml of AG-1X8 (100 to 200 mesh) in Bio-Rad Poly-Prep columns. Samples were loaded in 2 ml of 0.4 M HCl–1 M HF, and residual matrix elements were rinsed off with another 6 ml of 0.4 M HCl–1 M HF before Ti was collected with 7 ml of 6 M HCl.

Chromium was further purified following the methods outlined in (*85*, *86*), using 1 ml of AG50W-X8 (200 to 400 mesh) in Muromac Mini columns. After cleaning and conditioning, samples were loaded in 5 ml of 0.5 M HCl. Collection of Cr starts right away and continues with an additional 8 ml of 0.5 M HNO_3_. Nickel, Mg, Ca, and other elements were subsequently collected with 6 ml of 6 M HCl. The Cr cut was further purified using 0.25 ml of AG50W-X8 (200 to 400 mesh) resin in self-made transfer pipette columns. Samples were loaded in 3 ml of 0.5 M HNO_3_, and matrix elements were eluted with another 1 ml of 0.5 M HNO_3_, 3 ml of 0.5 M HF, and 6 ml of 1 M HCl before Cr was collected with 8 ml of 1.8 M HCl. This final column was repeated once to ensure removal of any remaining matrix elements from the Cr fraction.

After purification, all samples were fluxed with a 1:1 mixture of concentrated HNO_3_ and H_2_O_2_ at 130°C to decompose any residual organics from the resins before being converted to 0.5 M HNO_3_ (Fe and Cr) or 0.5 M HNO_3_–0.005 M HF (Ti). This purification procedure ensures high (>90%) yields and results in refined cuts with minimal impurities and negligible levels of isobaric interferences. Blanks were 1.7 ng for Fe, 2.1 ng for Ti, and 0.9 ng for Cr.

Isotopic compositions were measured following the methods outlined in (*80*). Iron isotope ratios were measured on a Thermo Fisher Scientific Neptune Plus MC-ICPMS using Jet and H cones in combination with an ESI Apex 2 IR at the LLNL. Measurements were performed in medium resolution mode on the low-mass flat shoulder plateau of the peak, resulting in signals of ∼250 V on ^56^Fe for Fe solutions containing 5 μg/g Fe. Isotope data were internally normalized to a ^57^Fe/^56^Fe ratio of 0.023095 (*79*). Mass 56 was collected with a Faraday cup connected to a 10^10^-Ω amplifier, and masses 53 and 60 were monitored with 10^12^-Ω amplifiers for potential interferences from Cr and Ni. All other masses were collected with 10^11^-Ω amplifiers. Measurements consisted of 100 cycles of 4 s each and resulted in an external reproducibility (2 SD) of approximately ±0.25 for ε^54^Fe and ±0.40 for ε^58^Fe as determined by repeat analyses of BCR-2 and BHVO-2 (Table 1). No on-peak baselines were collected. Samples were analyzed using sample-standard bracketing relative to the IRMM-524B Fe standard solution, and epsilon values are calculated relative to this standard.

Isotopic compositions of Ti were measured on the Thermo Fisher Scientific Neoma with Jet and X cones in combination with a Cetac Aridus II at the LLNL. Measurements were performed in medium resolution on the low-mass shoulder plateau of the peak, resulting in ∼40 V for ^48^Ti for Ti solutions containing 200 ng/g Ti. All masses were collected with Faraday cups connected to 10^11^-Ω amplifiers. Measurements were bracketed by the Origins Lab (OL-) Ti standard solution, and isotope data were normalized to a ^49^Ti/^47^Ti ratio of 0.749766 (*77*). Measurements consisted of 50 cycles each 8 s, resulting in external reproducibilities (2 SD) of approximately ±0.25 for ε^46^Ti, ±0.10 for ε^48^Ti, and ±0.25 for ε^50^Ti as determined by repeat analyses of the USGS rock standards BCR-2 and BHVO-2 (Table 1).

Isotopic compositions of Cr were measured on the Thermo Fisher Scientific Neoma with Jet and H cones in combination with a Cetac Aridus II at the LLNL. Measurements were performed in medium resolution on the low-mass shoulder plateau of the peak, resulting in ∼30 V for ^52^Cr when Cr solutions containing 600 ng/g Cr were introduced. All masses were collected with Faraday cups connected to 10^11^-Ω amplifiers. Measurements were bracketed by the SRM979 Cr standard solution, and isotope data were normalized to a ^50^Cr/^52^C ratio of 0.051859 (*78*). Measurements consisted of 50 cycles each 8 s, resulting in external reproducibilities of ±0.13 for ε ^53^Cr and ±0.25 for ε ^54^Cr as determined by repeat analyses of BCR-2 and BHVO-2 (Table 1).

### Reproducibility and accuracy of the isotope data

Many aliquots of the OREX samples were analyzed at ETH and LLNL for Ti, Cr, and Fe isotope data (Table 1), whereas Aquas Zarcas was analyzed for Ti and Fe isotopes in both laboratories. After digestion and aliquoting, all these samples were treated independently in the two laboratories using different chemical separation procedures and mass spectrometers as outlined above. The resulting Ti, Cr, and Fe isotope data obtained at the ETH and LLNL overlap with each other within the reported analytical uncertainties (Figs. 1 and 3 and Table 1). This demonstrates the robustness and accuracy of the data. For other samples (e.g., Orgueil), different sample chips were independently powdered and processed in both laboratories alongside the Bennu samples. These data indicate small inherent sample heterogeneities, e.g., for ε^50^Ti of Orgueil with values from 1.83 ± 0.07 to 2.03 ± 0.05 and ε^54^Cr values of 1.35 ± 0.12 to 1.46 ± 0.10 (Table 1).

### ^53^Mn-^53^Cr systematics of Bennu samples established by aqueous alteration

**Fig. 6.**
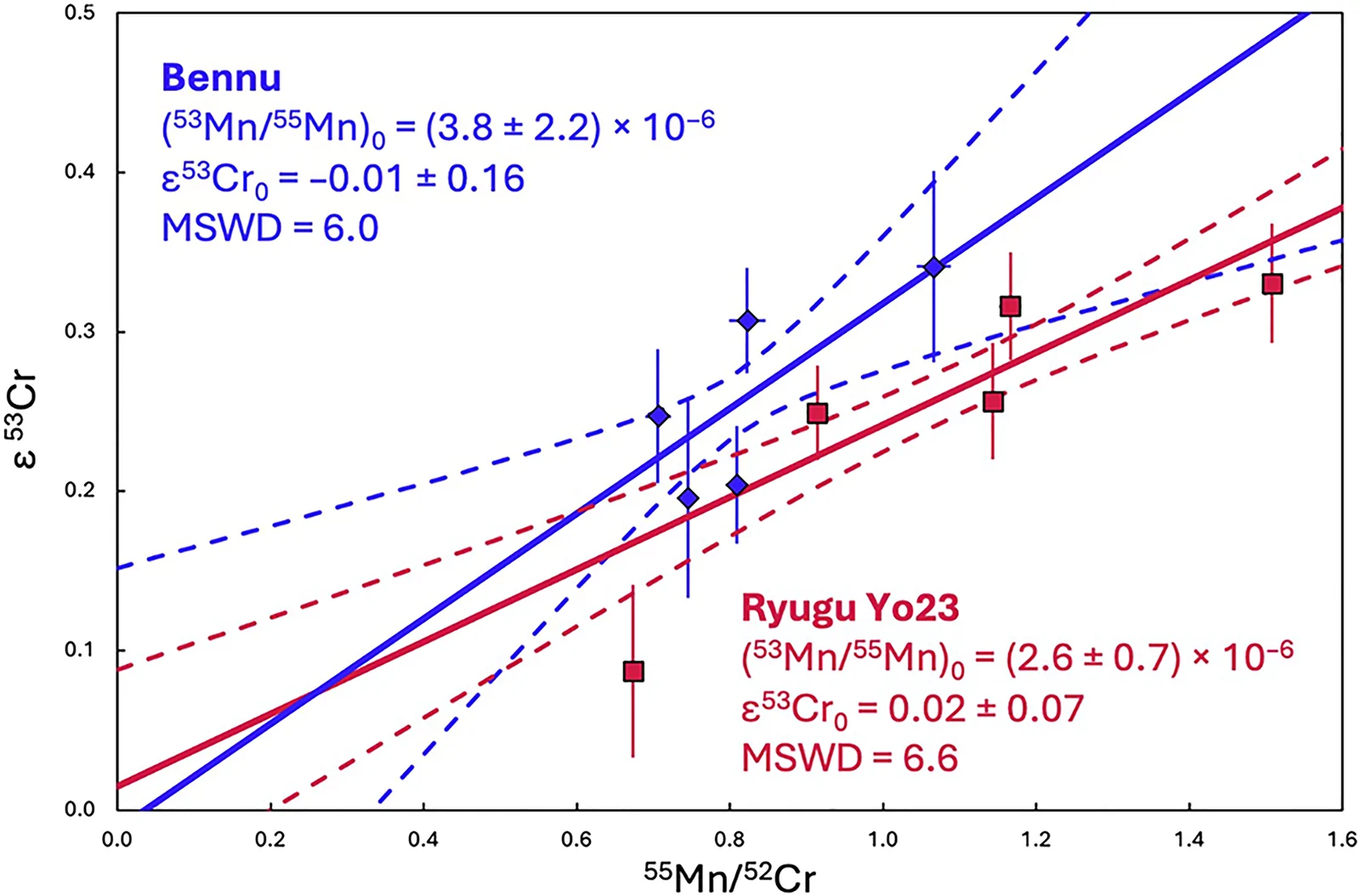
^53^Mn-^53^Cr isochron diagram of Bennu (blue diamonds) and Ryugu (red squares). The solid blue line and dashed curves represent a regression line for the five Bennu samples and the 95% confidence interval error envelopes, respectively, which were determined using IsoplotR (*93*). The same regression line is shown in red for the five Ryugu samples from (*3*, *6*). Bennu data are from Tables 1 and 2. MSWD, mean square weighted deviation.

The Mn/Cr ratios of the analyzed Bennu samples correlate with ε^53^Cr (Fig. 6) despite significant scatter (mean square weighted deviation of 6.0). The slope of the regression line through the Bennu data yields an initial ^53^Mn/^55^Mn ratio of (3.8 ± 2.2) × 10^−6^, which is higher than those of the Ryugu bulk samples [(2.6 ± 0.7) × 10^−6^ (*6*) and (3.12 ± 0.34) × 10^−6^ (*87*)], but overlaps within uncertainty. These ages likely date aqueous alteration on the first-generation parent body of Bennu and Ryugu. The scatter and the different initial abundances could result from carbonates that formed at different times during parent body alteration or from the inclusion of other components within the analyzed bulk samples that are not linked to carbonate precipitation (*6*).

The higher initial ^53^Mn/^55^Mn ratio hints that the Bennu ages on average are slightly older than those of Ryugu, although this difference is not fully resolved. Bennu data show less spread in Mn/Cr ratios than Ryugu data and as a consequence yield a less well-defined isochron. The Bennu average value for the initial ^53^Mn/^55^Mn ratio of 3.8 × 10^−6^ is closer to that of (*32*), a study that determined Mn-Cr isochrons on Ryugu dolomite using secondary ion mass spectrometry (SIMS) and determined an age of 2.0 to 2.9 Myr after CAI formation for the oldest carbonates, depending on the time anchor used [Pb-Pb age of D’Orbigny angrite (*88*) versus a statistical approach suggested by (*89*); see (*32*) for more details]. This emphasizes that the first-generation parent body of Bennu and Ryugu may have accreted before 2.0 to 2.9 Myr after CAI formation.

As for Ryugu samples (*6*), the Mn-Cr fractionation seems to be primarily controlled by the abundance of secondary carbonates. Evidence for this is provided by the correlation of CI-normalized Mn/Cr and Fe/Mn ratios for both bulk Bennu and Ryugu samples (Fig. 2A), as would be expected from variable dolomite abundances. Bennu samples show lower CI-normalized Mn/Cr and higher Fe/Mn ratios compared to most Ryugu samples, which indicates less dolomite in the analyzed Bennu samples compared to those of Ryugu and very close abundances to CI chondrites.
