# Supplementary material for: Nucleosynthetic constraints on the origin of Bennu and CI-like asteroids
Source: Sci Adv. 2026 Sep 23;12(39):eaei9107. doi: 10.1126/sciadv.aei9107 (PMC13600240; doi:10.1126/sciadv.aei9107)
Supplement: Supplementary file 1 [file sciadv.aei9107_sm.pdf]

Science Advances
AAAS

Supplementary Materials for

Nucleosynthetic constraints on the origin of Bennu and CI-like asteroids

Maria Schönbächler *et al.*

Corresponding author: Maria Schönbächler, mariasc@ethz.ch

Sci. Adv. **12**, eaei9107 (2026)
DOI: 10.1126/sciadv.aei9107

The PDF file includes:

Legends for data S1 to S7

Other Supplementary Material for this manuscript includes the following:

Data S1 to S7

Data S1-7. (separate excel file)

The file contains the tabulated data behind the figures.
Data Table 1: Group averages Ti, Fr, Cr, Zr, Ru and Nd isotope data from literature
Data Table 2: Compilation of Ti isotope data
Data Table 3: Compilation of Fe isotope data
Data Table 4: Compilation of Cr isotope data
Data Table 5: Compilation of Zr isotope data
Data Table 6: Compilation of Ru isotope data
Data Table 7: Compilation of Nd isotope data
